# Impact of modifiers on soil–water characteristics of graphite tailings

**DOI:** 10.1038/s41598-024-52826-6

**Published:** 2024-02-20

**Authors:** Changbo Du, Xinxin Lu, Fu Yi

**Affiliations:** 1 College of Civil Engineering, Liaoning Technical University, Fuxin, 123000 China; 2 Beijing Jingneng Geological Engineering Co., Ltd, Beijing, 102300 China

**Keywords:** Engineering, Materials science

## Abstract

To achieve integrated resource utilization of graphite tailings to improve their water-holding capacity, river silt and cow dung powder were added to graphite tailings as organic matter improvers. Improver ratios were designed using 4 g cow dung powder and 20, 30, and 50 g river silt. Soil–water characterization tests were performed using a combined tensiometer and filter paper method based on optimum density measurements. Analysis of the influence of river silt dosing on the soil–water characteristic curves of improved graphite tailing specimens was performed with data fitting using the Van Genuchten model. Here, we investigated the effect of river silt dosing on the internal pore structure and water-holding capacity of modified graphite tailing samples and verified the applicability of the model to graphite tailings. Our results demonstrate that the organic matter improver incorporated into graphite tailings can improve the internal structural compactness of graphite tailings, improving the water holding capacity. With an increase in river silt dosage, the saturated water content is larger, and the residual water content increases and then decreases. When river silt dosage is 30 g, the residual water content is the highest at a value of 3.32%. The van Genuchten model was highly accurate for assessing the graphite tailings. With an increase in river silt doping, the internal pore space first decreased and then increased, and the internal structure gradually became compact and loosened. The internal structure was in the optimal state in the experimental study when the dosage of cow dung powder was 4 g and the dosage of river silt was 30 g. The water holding capacity was optimal at this time. The results of this study provide a theoretical foundation for graphite-tailing-based mine reclamation and play a guiding role in exploring the value of the hydraulic characteristic index parameters when applying graphite tailings engineering.

## Introduction

The demand for graphite as a high-performance raw material has increased yearly^1,2^. Approximately 13 tons of graphite tailings are required for every 1 ton of purified graphite^3,4^. As graphite deposits are mined and the number of tailing dams gradually increases over time, the accumulation of graphite tailings exert an irreversible impact on the environment^5^. Currently, over 2000 ha of land in China is used for the discharge and storage of graphite tailings^6^. Graphite tailings can easily inhibit the survival of active enzymes in the soil^7^, ultimately causing damage to the physical and chemical structures of the environment and soil around the graphite tailings dam and restricting plant growth. Graphite tailings are fine-grained and prone to dust, and this can result in severe air pollution^8^. The main governance measures include physical engineering governance and repair methods^9^, chemical restoration means^10^, and bioremediation treatment means^11^. Graphite tailings can be utilized as (1) mine filling materials, (2) construction materials, (3) mining area soil and other soil improvement materials, and (4) components of industrial extraction to acquire valuable materials for secondary processing. Adhering to the construction of green mines reduces the impact of the development of graphite tailing resources on the ecological environment and achieves a win–win situation between social development and ecological environmental protection. In this study, we assessed graphite tailings soil improvement to achieve integrated resource utilization of graphite tailings.

Graphite tailings soil improvement is important for reducing waste of land resources, reducing land and air pollution, and optimizing the quality of the mine ecosystem^12^. The basic method for soil improvement of graphite tailings is vegetation restoration, and water is a prerequisite for plant growth For the improvement of water holding capacity, Shen^13^ applied organic amendments to sandy soil to promote the nutrient content of sandy soil, improve the internal structure of sandy soil that is loose and not cohesive, and improve the water holding capacity, all of which played a role in promoting plant growth. Sun et al.^7^ added multicomponent content of polyacrylamide (PAM) to iron tailings at low dosage concentrations and demonstrated that PAM exerted a significant improvement effect on the water holding capacity of iron tailings and improved the infiltration capacity of iron tailings. Mei^14^ and others reported that biochar helps to improve water holding capacity and that this capacity increases with increasing pyrolysis temperature. Zhang^15^ and others demonstrated that with the increase of biochar addition, the internal pore distribution changed significantly and formed a large number of macropores and mesopores. Additionally, the water-holding capacity was improved. Lv^16^ reported that the addition of PAM to iron tailings can improve the physical properties and water transport of iron tailings and create suitable living conditions for the recovery of vegetation in the soilless area. In summary, studies have demonstrated that the application of amendments can alter the internal pore structure and improve water transport, thereby increasing water holding capacity.

The soil–water characteristic curve (SWCC) can directly reflect the internal pore condition and water-holding capacity and is important for the study of internal water transport and retention. Currently, available studies have not been able to determine the relationship between matric suction and water content based on the basic physical and mechanical properties of the material. Therefore, research tools are primarily used to fit an empirical model with the experimental test results to obtain the SWCC. Commonly used empirical models include the Gardner^17^, Broods-Corey^18^, Van Genuchten^19^, Gardner-Russo^20^, and Fredlund and Xing^21^ models. To study the SWCC model, Liu^22^ proposed that the FX model possesses a high fitting accuracy for tailing SWCC and can better reflect the water-holding properties of tailings. Tang^23^ obtained the permeability function of graphite tailings by fitting and combining the Van Genuchten model based on the principle that the particle analysis curve is similar in shape to the SWCC. Chen^24^ proposed the Van Genuchten model to fit the relationship between the matric suction and water content of fine-grained tailings and to demonstrate the accuracy and applicability of the filter paper method experiments. In summary, the SWCC is an important relationship curve for studying the water-holding capacity of materials such as tailings. Current research focused on SWCC and the modelling of tailings primarily focuses on a single test method in combination with model fitting or the theoretical derivation of similar principles. In contrast, less research has been conducted to examine SWCC and the water-holding capacity of graphite tailings through a combined experimental method and a model fitting approach.

In this paper, adhering to the concept of "waste for waste,” common river sludge and cow dung powder are added to graphite tailings as organic matter improver. Considering the drawbacks of a single test method, the SWCC of graphite tailings under different river silt doping conditions was measured using a combined tensiometer and filter paper method. The data were fitted using the Van Genuchten model to investigate the effect of river silt doping on the water-holding capacity of the graphite tailings and to validate the applicability of the model.

## Materials and methods

### Test materials and programs

**Figure 1:**
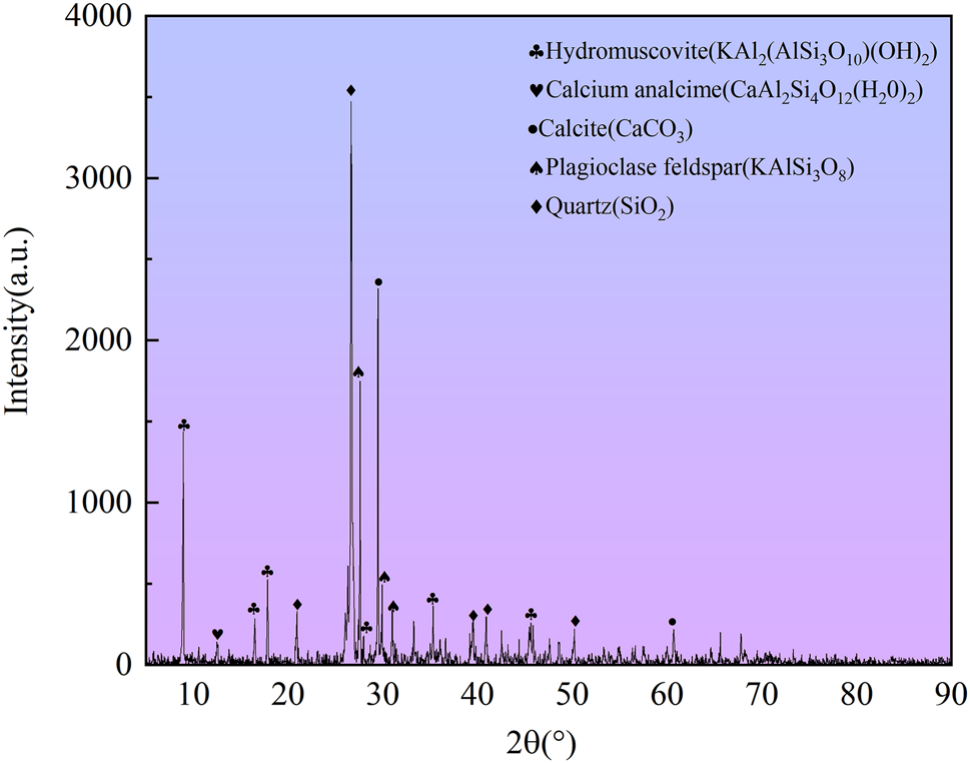
X-ray diffraction pattern of graphite tailings.

Graphite tailings were obtained from a graphite mine reservoir in Luobei County, Jixi City, Heilongjiang Province, China. The samples underwent x-ray fluorescence (XRF) and x-ray diffraction (XRD) testing and analysis. An X-ray diffractometer with a 2θ angle (0–90°) was used to scan the entire diffraction area as an angle change and to assess the abscissa of the X-ray diffraction spectrum. The intensity of diffraction peaks at different diffraction angles is taken as the ordinate. We obtained the elemental (Table 1) and mineral composition of graphite tailings (Fig. 1). The XRF chemical analysis of graphite tailings samples in Table 1 reveals that SiO_2_ is the primary chemical component of graphite tailings with a content of 51.163%. Graphite tailings also contain high levels of Al_2_O_3_, CaO, and Fe_2_O_3_ and small amounts of SO_3_, K_2_O, and TiO_2_.

**Table 1:** X-ray fluorescence analysis of graphite tailings.

Oxide	Contents (%)
SiO_2_	51.163
Al_2_O_3_	13.305
CaO	13.342
Fe_2_O_3_	8.092
SO_3_	6.732
K_2_O	3.844
TiO_2_	0.857

The XRD mineral composition analysis of the graphite tailing samples (Fig. 1) demonstrated that quartz was the predominant component, with hydrous muscovite, calcite, plagioclase zeolite, and plagioclase microfeldspar present in lesser quantities.

The basic physical parameters of the graphite tailing samples are listed in Table 2. According to the data presented in the table, the porosity of graphite tailings is 41.53%. The natural dry density was 1.613g/cm^3^. The permeability coefficient is 1.101 × 10^−4^cm/s. Based on the classification of sand, it can be classified as semipermeable. The pH of the graphite tailings was 8.19 and is slightly alkaline).

**Table 2:** Physical parameters table of graphite tailings.

Loose bulk density (g/cm^3^)	Tight packing density (g/cm^3^)	Density (g/cm^3^)	Void rate(%)	Maximum dry density(g/cm^3^)	Maximum dry density(g/cm^3^)	Natural dry density(g/cm^3^)^3^	Permeability coefficient	pH
1.39	1.71	2.38	41.53	1.95	1.41	1.61	1.10 × 10^−4^	8.19

**Figure 2:**
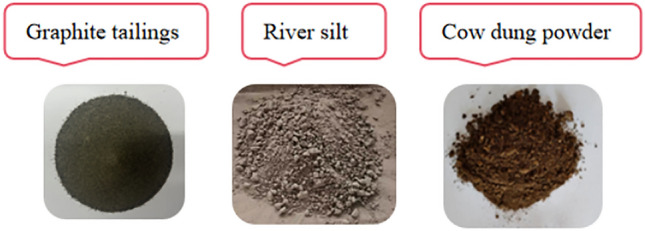
Test material.

Organic matter improvers can enhance the structure of agglomerates, increase the number of capillary pores, and improve water absorption and water-holding capacity. The graphite tailings and organic matter amendments (river silt and cow dung powder) are presented in Fig. 2. River silt was obtained from the Xihe District, Fuxin City, Liaoning Province. Cow dung powder was obtained from cattle farms in Xinxiang City, Henan Province, China. The application of river silt to graphite tailings not only solves the problem of river silt elimination but also improves the structure of agglomerates within the graphite tailings. Cow dung powder contains a large amount of undecomposed plant fibers that can be continuously decomposed into a stable organic carbon source when added to the graphite tailing samples. The ability to increase the organic carbon storage of the modified graphite tailing samples and improve the agglomeration of the modified graphite tailing samples enhanced the internal agglomerate structure. Existing research has demonstrated that when the mud and sand doping ratios are 30–50%, the sludge content is too high and water cannot easily infiltrate. The composite media absorption rate is also reduced^25^. The application of 2.5% alone and 5% chicken manure exerted the same ameliorative effect on rare earth mine tailings^26^. Considering the practical application value and the ecological characteristics, this can allow for graphite tailings to be converted between solid and rheological states after the addition of improvers. The particle arrangement of the overall structure remained unchanged, and thus, it possessed a certain self-healing ability. Ultimately in this study, an improved ratio design was selected that included cow dung powder dosed at 4g and river silt dosed at 20, 30, and 50g. The material ratios are presented in Table 3. The first group was the control group, and the remaining groups were the test groups.

**Table 3:** Material ratio of graphite tailings matrix soil.

	Graphite tailings (g)	River silt (g)	Cow dung powder (g)
Group 1	50	0	0
Group 4	50	20	4
Group 3	50	30	4
Group 2	50	50	4

**Figure 3:**
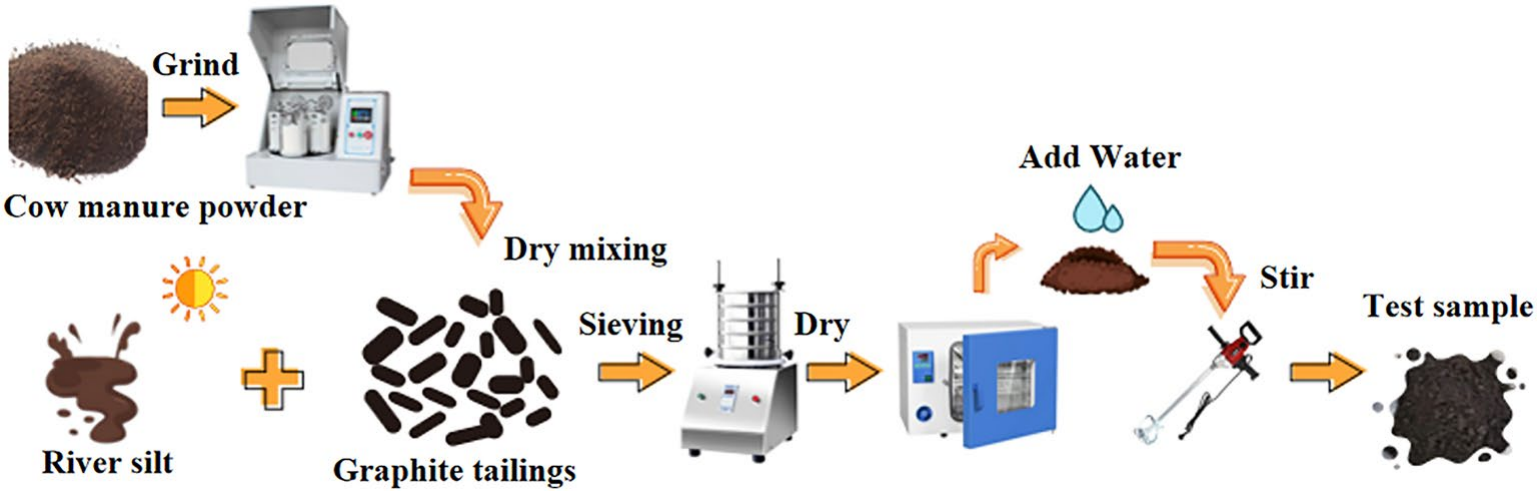
Flow chart of the preparation of graphite tailings matrix soil.

Factors such as particle size, doping rate, and the high-water absorption capacity of river silt may affect the quality of the modified graphite tailing specimens. Therefore, when preparing the reshaped soil, the cow manure powder was ground using a planetary ball mill to ensure uniform particle fineness and then passed through a 1 cm round hole sieve. Graphite tailings and river silt were naturally dried and then sieved through a 1 cm round hole sieve. The method of “drying first and then wetting” was employed to avoid uneven humidity of the material. Finally, the mixture was thoroughly mixed at 2600 (r/min) to obtain the graphite tailing matrix. Random sampling was used to produce materials suitable for indoor testing (Fig. 3).

**Figure 4:**
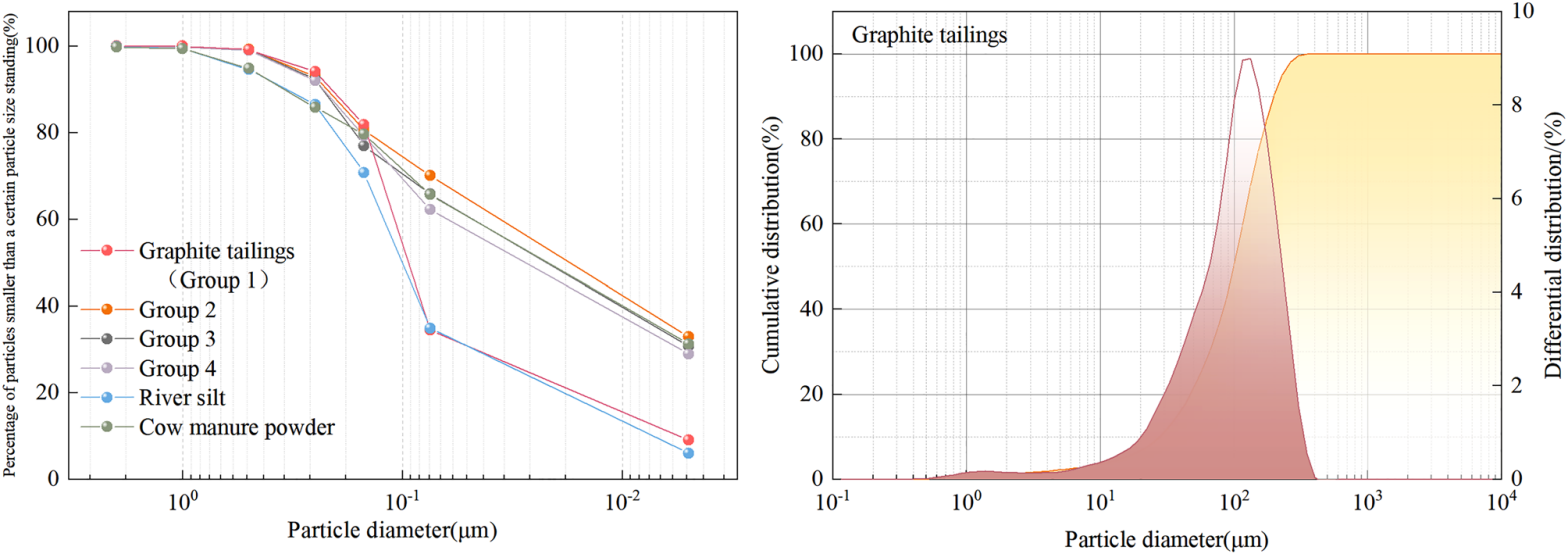
Particle grading curve.

After the preparation of the modified graphite tailing specimens, particle gradation curves were obtained using a motorized sieving machine for the different river silt admixtures (Fig. 4). The curvature and inhomogeneity coefficients are presented (Table 4). Figure 4 indicates that a higher river silt content results in a smoother curve and better grading. All graphite tailing subsoil grades were good after application of the amendments. At this point, the internal pores of the graphite tailings were filled with finer particles, thus resulting in a denser state.

**Table 4:** Graduation analysis.

	Group 1	Group 2	Group 3	Group 4
D_10_	0.086	0.046	0.038	0.032
D_30_	0.164	0.126	0.113	0.099
D_50_	0.242	0.203	0.189	0.176
D_60_	0.301	0.241	0.228	0.214
C_c_	1.039	1.432	1.473	1.431
C_u_	3.500	5.240	6.000	6.688

### Test principle

The tensiometer method measures a limited range of substrate suction, but it is easy to obtain values of substrate suction for the critical saturated moisture content. The filter paper method can be used to measure the full suction range; however, it is more difficult to operate in the critical saturation state. Therefore, this study adopted a tensiometer and filter paper coupling method to conduct soil–water property tests.

#### Tensiometer method

A tensiometer is a device that can directly measure the suction force of a substrate and is primarily composed of a ceramic head with a high air entry value and a pressure measurement system. The water-saturated ceramic head was in good contact with the modified graphite tailing samples. Due to the unique air barrier and water conductivity of the ceramic, the negative pore water pressure in the modified graphite tailings specimens could be removed from the tensiometer through the ceramic head, thus forming a negative pressure in the tensiometer. At each equilibrium point, the water in the tensiometer was sampled in the pore drying water with the same pressure through a vacuum gauge connected to the tensiometer or negative pressure sensor to measure the suction force in the soil.

#### Filter paper method

The filter paper method was used to indirectly measure the suction of the modified graphite tailing specimens. It is widely used because of its low price, ease of handling, full suction range, and accurate test results. In this method, dry filter paper was placed in the middle of the modified graphite tailing specimens. The filter paper absorbed water from the modified graphite tailing specimens via capillary action until the moisture in the modified graphite tailing specimens and the filter paper reached equilibrium.

The filter paper used in this study was made of domestic “Double Circle” brand quantitative filter paper. The filter paper is available in slow (No. 203), medium (No. 202), and fast (No. 201) versions^27^. The pattern of the SWCCs obtained in experiments using Whatman's No. 42 filter paper and the “Double Circle” brand No. 203 filter paper are similar, and both materials provide accurate and reliable data. Therefore, this study used the “Double Circle” brand No. 203 quantitative filter paper produced by Hangzhou Xinhua Paper Factory as the test filter paper. The main technical specifications of the No. 203 filter paper are: 70 mm diameter; slow filtration speed; 0.01% ash content. Qualitative filter paper must be placed on the top and bottom of the quantitative filter paper to prevent the modified graphite tailing specimens from sticking to the filter paper. The qualitative filter paper used in this study was “Liu Xin” brand No. 102 qualitative filter paper produced by Jiangsu Liu Xin Science and Education Equipment Co. The main technical specifications of filter paper No. 102 are: 70 mm diameter; medium filtration speed; 0.15% ash content.

Many studies in China since 2000 have demonstrated that the same rate equation can be used to calculate the results of tests using the same type of “Double Circle” filter paper. The rate equation in this study was calculated using Eq. (1) ^28^ in a 200 kPa pressure membrane extractor of type 1250 as follows:

1 $$\lg \mu_{s} = \left\{ \begin{gathered} - 0.0767\omega + 5.493,\omega \le 47 \hfill \\ - 0.0120\omega + 2.470,\omega > 47 \hfill \\ \end{gathered} \right.$$

where *μ*_s_ is the matrix suction (kPa) and *ω* is the experimentally measured water content of the filter paper (%).

### Test method and procedures

#### Optimal density

The maximum water content was determined in relation to the density by performing a capillary pore water absorption test to determine the optimum density of the specimens. Graphite tailings subsoil indoor test samples are prepared and then dried (105 ℃ for 8 continuous h). The specimens with different densities were loaded into a ring knife measuring 61.8 (mm) × 20 (mm) at different densities and compacted into layers. The prepared ring-knife sample was placed in a Petri dish inside a glass container and separated using a filter paper. The interior of the glass container was filled with water so that the surface was slightly below the Petri dish. The sample was left to stand for 8 h to allow it to fully absorb the water via capillary. After measuring the sample mass, the sample was dried in a drying oven at 105 ℃ for 8 h. Subsequently, the moisture content was calculated. Two parallel tests were established for each group, and the average was calculated.

#### Tensiometer method

##### Tensiometer method

The test range included saturated moisture content to substrate suction with moisture content greater than 20%. The testing method and process of the tension meter method were as follows.

**Figure 5:**
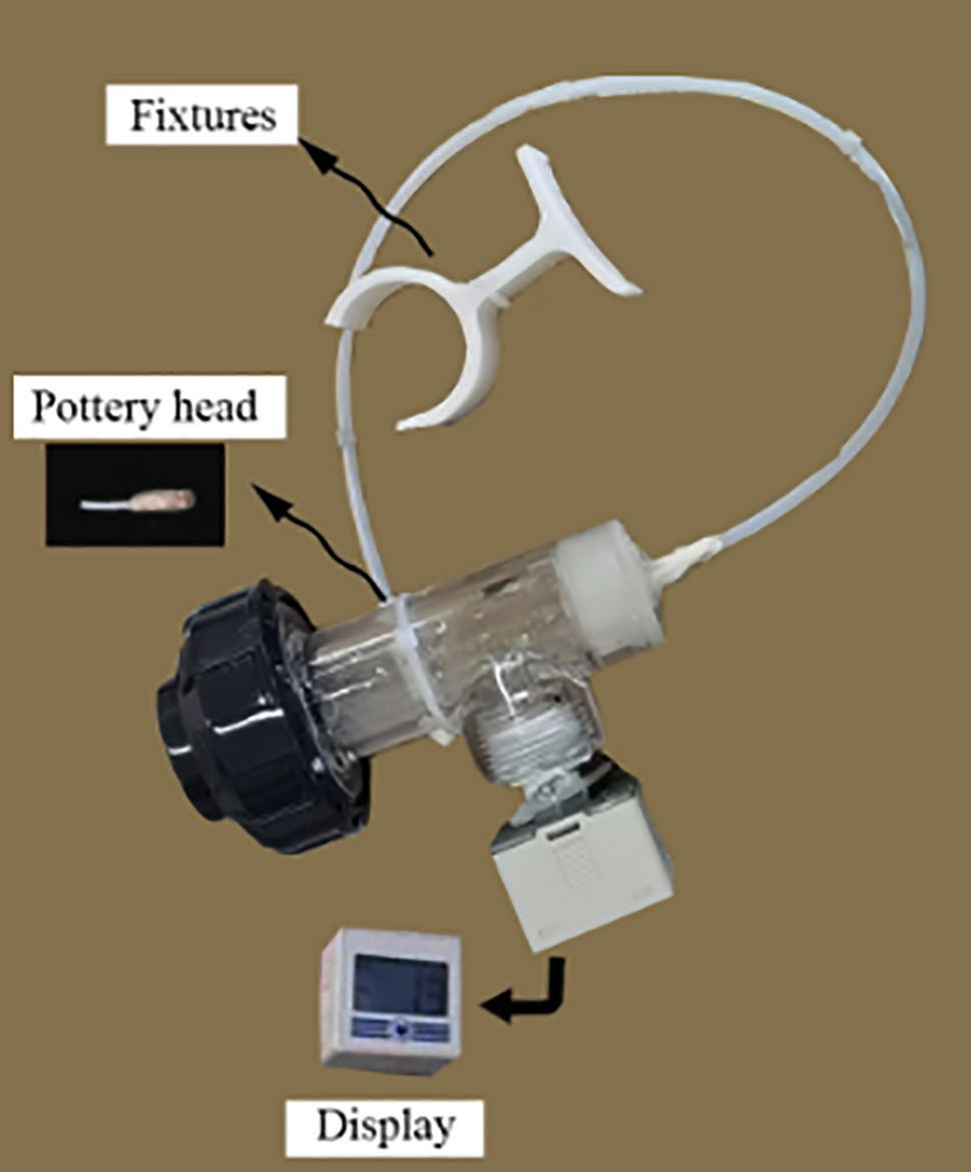
Tensiometer.

**Figure 6:**
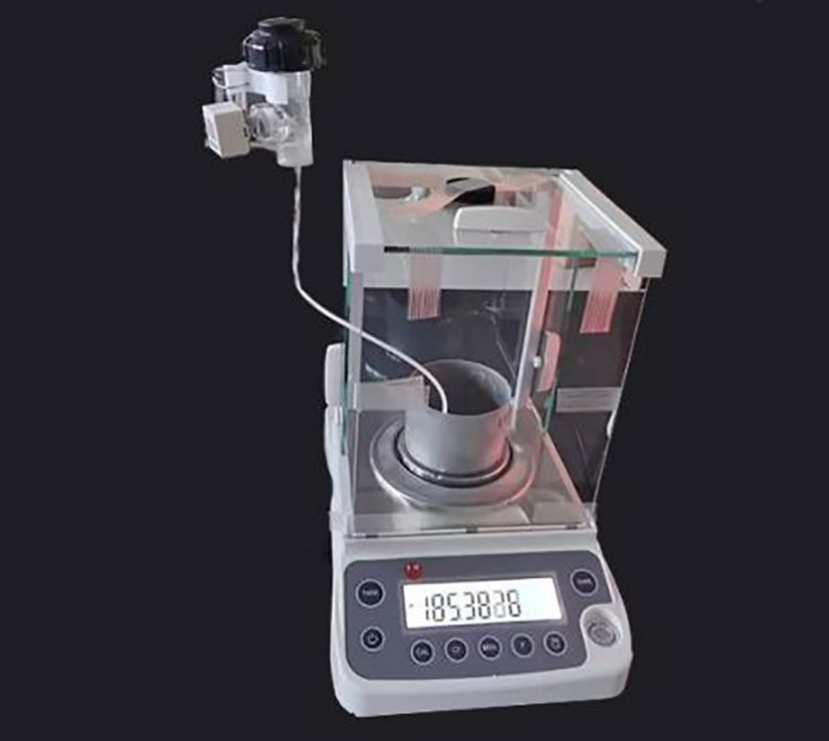
Test diagram of tensimeter method.

1. Graphite tailing specimens were modified in accordance with test requirements. We prepared ring knife specimens with reference to the < Standard for Unsaturated Geotechnical Test Methods > .
2. We caused the modified graphite tailings specimens to fully absorb water through capillary action through the ring knife method.
3. Commission and installed fixtures are presented (Fig. 5). A porous ceramic head was placed on the soil sample to ensure close contact (Fig. 6). Care was taken to avoid disturbances in the soil sample and to avoid affecting the testing accuracy. Tensiometer and electronic balance readings were recorded at 2 h intervals.

##### Filter paper method

The scope of the test included suction of substrates up to 20% moisture content. A set of samples was prepared at 2% moisture content intervals. The procedure for the filter paper method was as follows.

**Figure 7:**
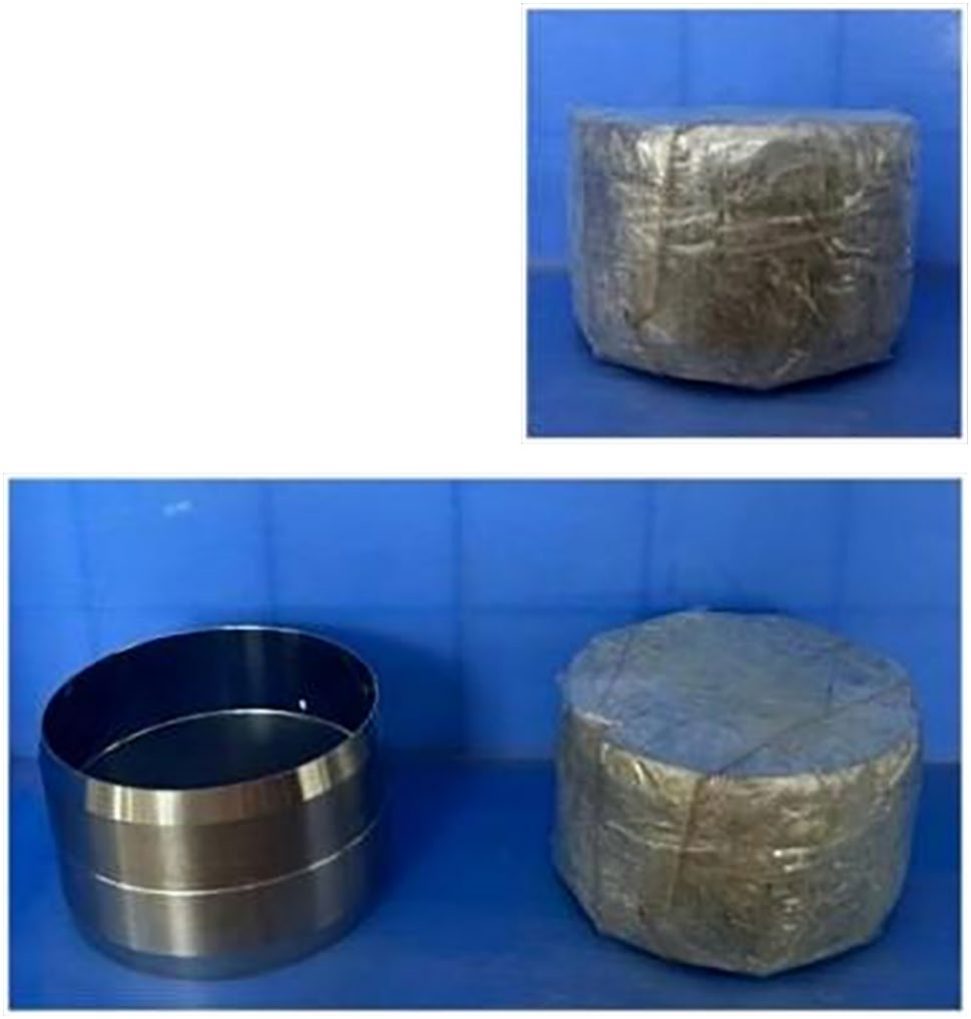
Filter paper sample.

1. We cut the filter paper required for the test appropriately. We cut it to ring knife size and then dried and stored the sample in a drying oven.
2. Graphite tailing specimens were modified according to the test requirements. When preparing the specimens, care was taken to keep the ring knife blade down to ensure a flat surface in contact with the filter paper.
3. We used tweezers to take the qualitative and quantitative filter papers and place them into the middle of the two ring knife specimens. The ring-knife specimens were wrapped tightly in a cling film to ensure that the specimens were well sealed (Fig. 7).

4) We sealed and stored the sealed ring knife sample at a constant temperature with a suction balance time of at least 7 d.

5) Once the moisture was equilibrated, the central filter paper was quickly removed using tweezers. The filter paper mass was measured using a high-precision electron balance. This step was completed within 3–5 s.

6) The wet filter paper was dried in an oven. The moisture content of the filter paper was calculated and substituted into Eq. (1) to calculate the matric suction at the moisture content of the modified graphite tailing specimens.

## Results

### Optimal density

**Figure 8:**
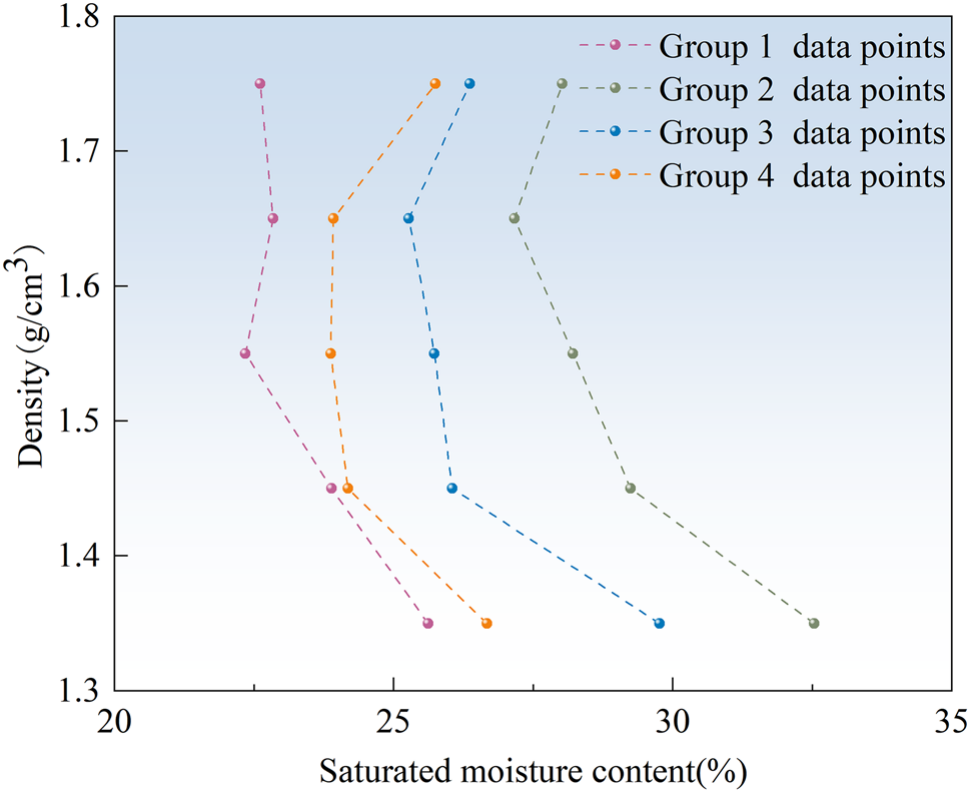
Test curve data points and fitting diagram.

The experimental data were obtained using density as the horizontal coordinate, maximum moisture content as the vertical coordinate, and the final test results (Fig. 8). Quadratic fitting was performed on the obtained data. A fitted curve was obtained as presented in Fig. 8. The optimum density of the graphite tailings was 1.65 g/cm^3^, as obtained by fitting the curve. Optimum densities of 1.64, 1.61, and 1.57 g/cm^3^ were determined for river silt at 50, 30, and 20g, respectively.

### SWCC

**Figure 9:**
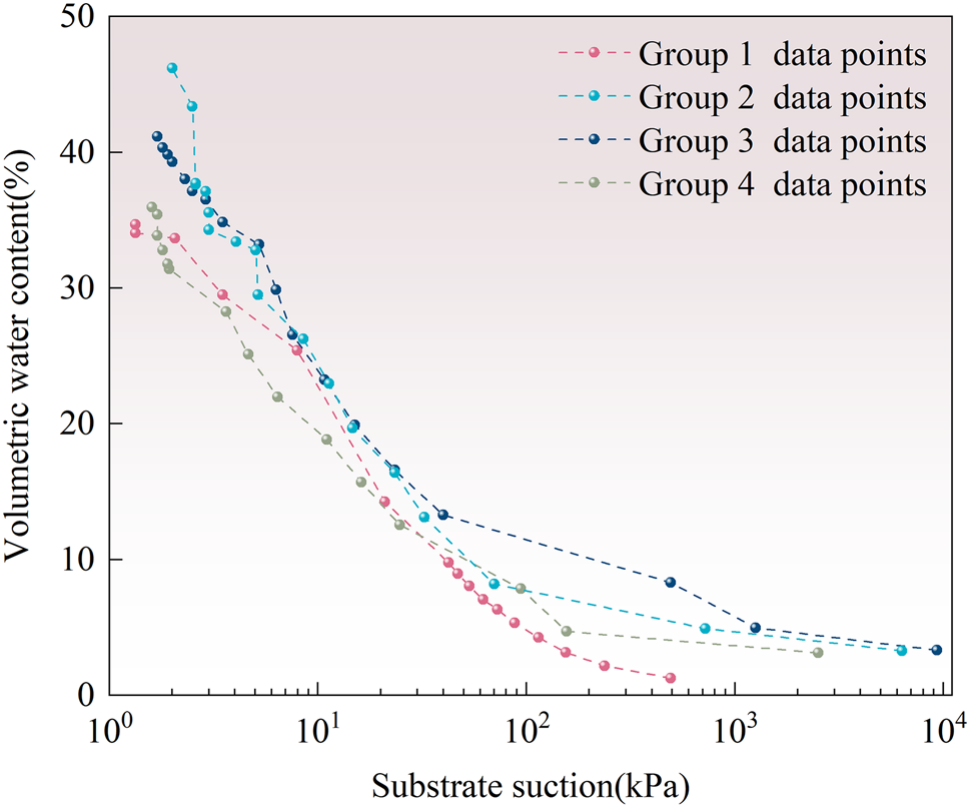
Results of soil–water characteristics test data.

The relationship curve between SWCC (water content and matric suction) of the graphite tailing specimens before and after modification was obtained based on the combination of the tensiometer and filter paper, and the results are presented in Fig. 9. Figure 9 provides the SWCC results of the graphite tailing specimens with different river silt dosages, and the data obtained from the tests are plotted and visualized. Observing the distribution pattern of data points, the SWCC dehumidification process of the modified graphite tailings specimens can be roughly divided into four stages that include the boundary effect, major transition, minor transition, and residual stages.

**Figure 10:**
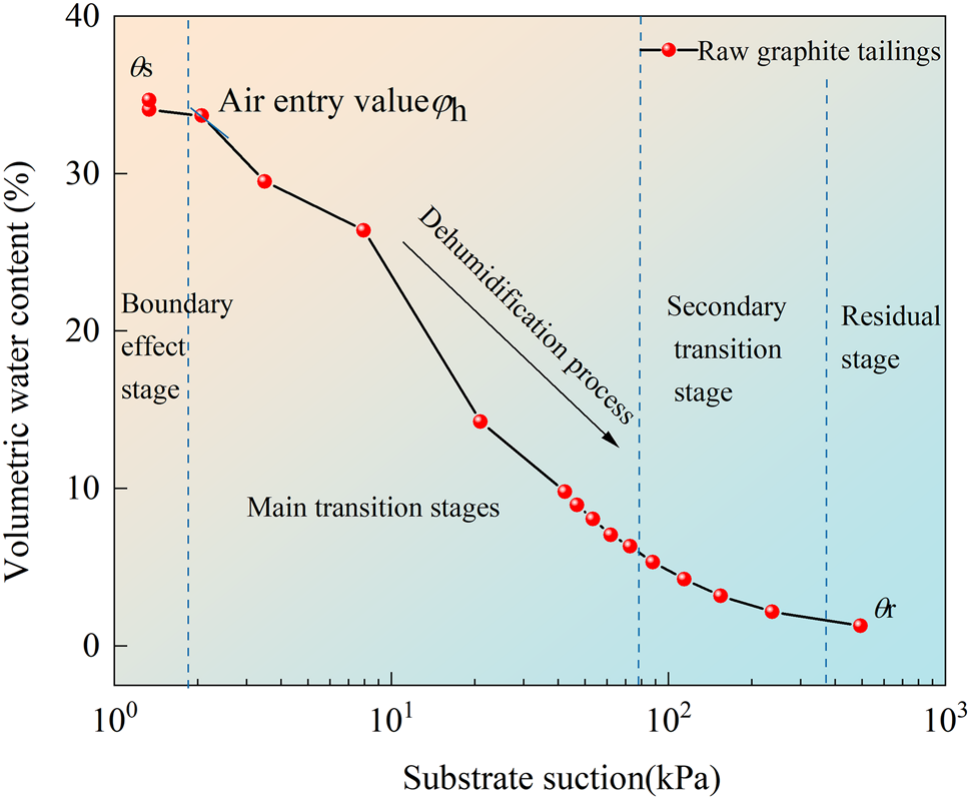
Diagram of dehumidification stages.

Using graphite tailings as an example, the dewetting process was divided into four stages (Fig. 10). In the first stage, the volumetric water content is close to the saturated volumetric water content. This stage is termed the boundary effect stage. At this point, the pores between the particles of the modified graphite tailing specimens were overwhelmingly filled with water. There was a change in the matrix suction but no change in the effective stress within the specimens. This also results in an essentially constant volumetric moisture content. The second stage is the main transition stage in which the volumetric moisture content decreases rapidly, and there is a clear downward trend in the curve. The third stage is the secondary transition stage in which the volumetric moisture content decreases slowly, and the curve tends to decrease more slowly. The second and third stages indicate that the substrate suction increases to the air entry value at this point. The gradual entry of air into the pores between the particles of the modified graphite tailing specimens resulted in the removal of water from the pores. At this point, the volumetric water content decreases rapidly as the suction of the substrate increases. As the pore space was gradually filled with air, the curve tended to decrease sharply and slowly. The fourth stage is the residual stage, where the volumetric moisture content approaches the residual volumetric moisture content and the curve tends to flatten. At this point, the pore water within the modified graphite tailings specimens existed only in small discontinuous pores, and the variations in the matric suction and volumetric water content both stabilized.

The experimental results presented in Fig. 9 indicate that the experimental curves exhibit the same pattern of change. During dehumidification, the matric suction increased as the volumetric moisture content decreased. Air entered between the boundary-effect stage and the main transition section. The air entry value is the theoretical value of the difference between the water and air pressures necessary for the maximum pore size of the modified graphite tailing specimens to generate suction. Therefore, it can be used as a parameter to measure the maximum pore size of the modified graphite tailing specimens.

The saturated water content increases as more river silt is added to the system. The curves for 50 and 30g river silt doping in the main transition phase lie above the curve for the original graphite tailings. The curve for 20g river silt is below the curve for the original graphite tailings. Upon entering the secondary transition phase, the curve for 30g river-silt doping was completely above that of the other test groups. In the secondary transition phase, when the matric suction was 100 kPa the maximum volumetric water content was 11.59% for 30g river silt, 7.59% for 50g, 7.17% at 20g, and 4.61% at 4.61% volumetric water content in the raw graphite tailings. Compared to the original graphite tailings, the volumetric water content increased by 39.8% with 30g river silt doping. Improved water-holding capacity of 30g river silt doped specimens under the same matric suction. The change in river silt dosage affects the soil–water properties of the specimens, and concurrently, the water holding capacity exhibits a trend of increasing and then decreasing with the increase of river silt dosage.

## Discussion

### SWCC model

The SWCC reflects the relationship between moisture content and substrate suction. The above test provided a line graph of the water content and substrate suction. The points in the graph represent discrete points. In theoretical studies and in practical applications, it is necessary to present the experimental results as a continuous function and to apply mathematical models to fit the data points for theoretical analysis. In this study, the Van Genuchten model was selected for data fitting.

#### Van Genuchten equation

The Van Genuchten equation^19^ is a function of the volumetric water content of the soil and matric suction as follows:

2 $$\theta = \frac{{\left( {\theta_{{\text{s}}} - \theta_{r} } \right)}}{{[1 + (\phi /\alpha )^{n} ]^{m} }} + \theta_{r}$$

3 $$S = \frac{1}{{[1 + \left( {\phi /\alpha } \right)^{n} ]^{m} }}$$

where *θ*_s_ the volumetric water content (%); *φ* is the matrix suction (kPa); *θ*_s_ is the saturated volumetric water content (%); *θ*_r_ is the residual water content (%);* S* is the saturation (%); *α* is the parameter that approximately corresponds to the air entry value; *m* and *n* are the curve trait parameters; *m* is related to the asymmetry of the soil sample model; *n* is related to the pore size distribution of the soil sample.

The parameters in the formula are calculated as follows:

When *φ* = *α*:

4 $$\theta_{{\text{q}}} = 2^{ - m} \left( {\theta_{{\text{s}}} - \theta_{r} } \right) + \theta_{r}$$

where *θ*_q_ is the volumetric water content (%) corresponding to the time at which the air entry value was reached.

The volumetric water content corresponding to the air entry value was equal to the volumetric water content of the capillary.

5 $$\theta_{{\text{q}}} = \theta_{m}$$

where *θ*_m_ is the moisture content in the capillary volume. This refers to the maximum amount of capillary-rising water that can be maintained within the soil mass.

6 $$\theta_{{\text{m}}} = 2^{ - m} \left( {\theta_{{\text{s}}} - \theta_{r} } \right) + \theta_{r}$$

The formula for *m* is as follows:

7 $$m = \frac{{\ln \left( {\theta_{{\text{s}}} - \theta_{r} } \right) - \ln \left( {\theta_{{\text{m}}} - \theta_{r} } \right)}}{{{\text{ln2}}}}$$

The equations for parameters *n* and *m* in the Van Genuchten equation are as follows:

8 $$n = 1 - \frac{1}{m}$$

#### Model applicability

**Figure 11:**
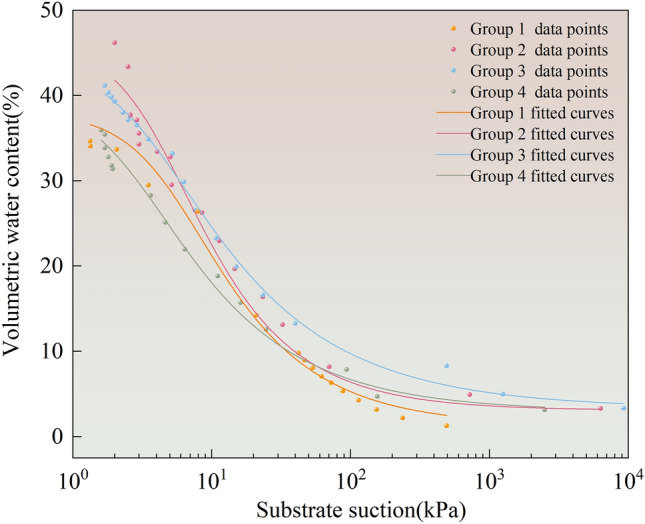
Soil–water characteristic curve (SWCC) is fitted.

The test data were curve-fitted using the Van Genuchten model. The fitting results are presented in Fig. 11. The parameters and goodness-of-fit obtained after fitting the Van Genuchten model to the experimental data are listed in Table 5.

**Table 5:** Soil–water characteristic curve fitting parameter table.

	Number of data point	m	n	α	θs (%)	θr (%)	R^2^
Group 1	16	0.428	1.747	5.183	37.96	1.27	0.99
Group 2	15	0.384	1.623	3.087	39.87	2.95	0.97
Group 3	18	0.353	1.546	3.354	44.29	3.32	0.99
Group 4	18	0.445	1.803	4.081	46.27	3.08	0.97

The fitted curves presented in Fig. 11 all exhibit an inverted “S” shape. From the values in Table 5, it can be observed that the correlation coefficients (R^2^) of the curve fits in Fig. 11 are not less than 0.97. This also indicates that the curve fit was good and highly accurate. A comprehensive analysis of the fitted curves revealed that the fitted curves for raw graphite tailings and river silt dosed at 20g were relatively similar. The fitted curves for the tests with 30g river silt dosing consistently possessed higher volumetric water content than the other test groups under equal substrate suction after entering the main transition phase. The application of organic matter amendments affected the water-holding capacity of graphite tailings that increased and then decreased as the silt content of the river increased. At the transition stage, the portion of water discharged by the matric suction was free water in the specimens, whereas the water discharged at the residual stage was bound water in the specimens. The residual moisture content tended to increase and then decrease with increasing river silt doping. The highest residual moisture content of 3.32% was observed at 30g of river silt doping.

### Analysis of fitting results

**Figure 12:**
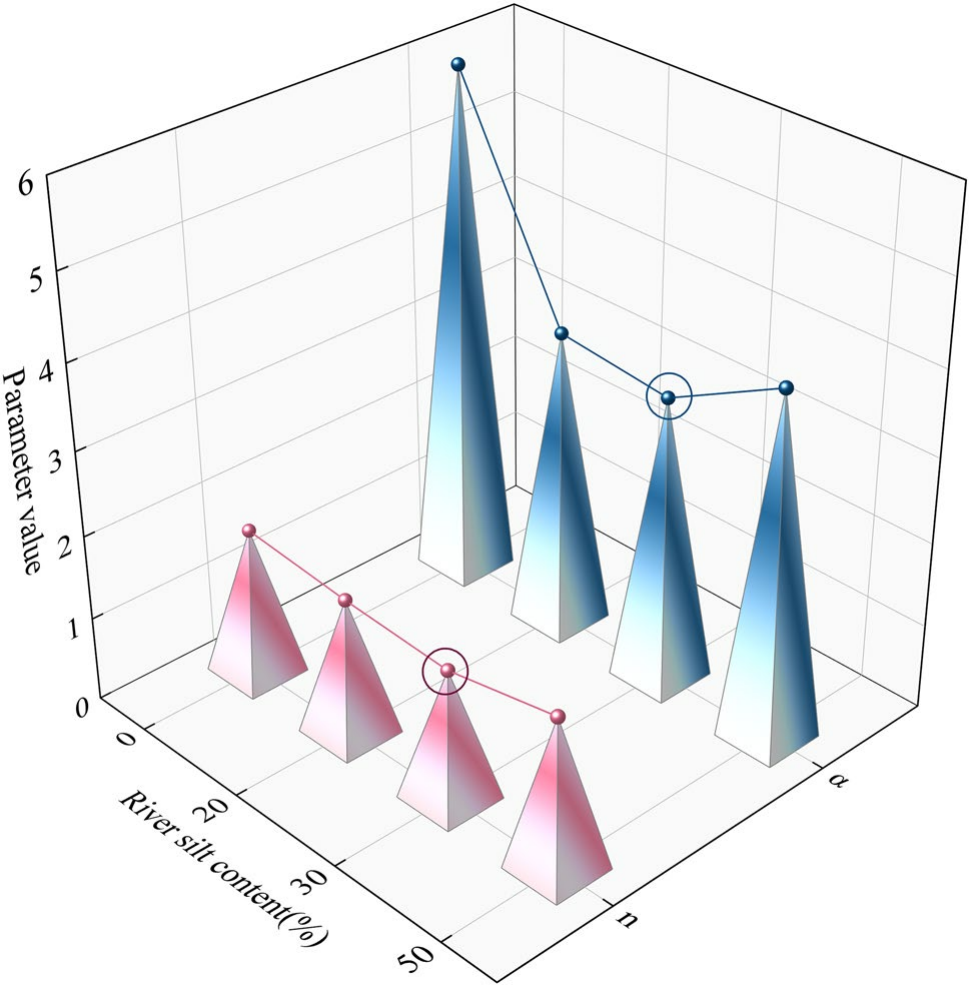
Change in fitting parameters with varied silt content.

There are two important parameters in the fitting results (parameter *n* and *α*). The curve fitting *n* value indicates the steepness of the curve. The larger the value of *n*, the steeper the fitted curve, and the more uniform the distribution of pores. The value of *n* presented in Table 5 exhibits a trend of first decreasing and then increasing with the increase in river silt content as presented in Fig. 12. According to the fitting results, the addition of river silt and cow manure powder caused structural changes in the graphite tailings. It was internally condensed into aggregate structures, ultimately resulting in an increase in small aggregate structures within the modified graphite tailing specimens, a decrease in the number of pores and pore size. Moreover, most of the internal pore volume was occupied by gas, and the water in the pores was isolated by the gas and modified graphite tailing specimen particles in the pores, thus resulting in a non-connected state. At this point, the water in the pores adhered to the surface of the modified graphite tailing specimen particles in the form of thin-film water, thus making it difficult for the skinny film water in the pores to be discharged and making it more difficult for the sample to lose water. Stronger water-holding capacity of modified graphite tailing specimens was observed. However, when the amount of river silt added increased to 50g, the water holding capacity is relatively weakened.

The curve fitting parameter *α* is the parameter associated with the air entry value. The air entry value is the suction value corresponding to the time at which air enters, and water is discharged from the largest pores of the modified graphite tailing specimens. The air entry value was the suction value corresponding to the intersection of the tangent line of the inflection point of the curve and the horizontal line of the saturated moisture content. The air entry value is a sudden change point on the SWCC. Before the air entry value is reached, the change in water content is almost negligible. After the matric suction reaches the air entry value, the water content decreases rapidly with increasing suction, and at this point, the gravitational water discharge dominates and the pore air intake (i.e., desaturation) develops rapidly. In Table 5, the *α* value decreases and then increases with the increase of river silt doping, and the air entry value increases and then decreases. With the increase of river silt doping, the number of large pores inside the modified graphite tailings specimens decreases and then increases, and when the river silt doping reaches 30g, the α-value increases. It can be observed that the internal structure is in the best state when the river silt doping is 30g.

The first inflection point of the fitted curve is the air entry value that is the dividing point between the boundary effect and main transition phases. The logarithmic value of the volumetric water content of the specimens was derived based on the suction of the matrix. We set the second-order derivative to be zero. The matric suction at the inflection point of the curve corresponds to the air entry value. The resulting equation is as follows:

9 $$\phi_{{\text{h}}} = \alpha \left( \frac{1}{m} \right)^{\frac{1}{n}}$$

The parameter values were incorporated into formula 9. The results of the calculations presented in Table 6 reveal that when reaches the air entry value, the volumetric water content first increases and then decreases with an increase in the silt content in the river channel and is greater than the volumetric water content of the graphite tailings before modification. This demonstrates that applying river silt and cow dung powder increased the moisture retention ability of the graphite tailing subsoil. When the air entry value was reached, the maximum pore size of the specimen was filled with air. As the amount of river silt doping increased, the pore sizes of the specimens initially decreased and then increased. The internal structure gradually became denser and peaked before loosening.

**Table 6:** Soil–water characteristic curve eigenvalue calculation results.

	Substrate suction when air entry value is reached *φ*_h_ (kPa)	Volumetric water content when reaching air entry value *θ*_q_ (%)
Group 1	5.740	23.180
Group 2	6.299	25.519
Group 3	6.578	28.817
Group 4	6.392	28.648

The calculated *θ*_q_ value provides a clear indication of where the modified graphite tailings specimens enters the transition phase. From Fig. 11, it can be observed that the modified graphite tailing specimens in the process of dehumidification are apparent when entering the transition stage after the river silt dosaged at 30g; the curve is always located above the other test group curve. After entering the second stage, the volumetric water content of graphite tailings with 30g river silt dosing was higher than the other test groups under the same conditions of substrate suction. This demonstrated that the water holding capacity is optimum at 4g dosage of cow dung powder and 30g dosage of river silt within the study area of this paper.

## Conclusions

An organic matter improver is added to graphite tailings to improve the water-holding capacity of graphite tailings. And to investigate the effect of river silt admixture in improvers on the water-holding capacity of graphite tailings. Soil–water characterisation of improved graphite tailings using a combined tensiometer and filter paper method at different river silt dosages. Fitting of experimental data using the Van Genuchten model to investigate the effect of river silt dosage on the internal pore structure and water-holding capacity of graphite tailings. The following conclusions were drawn from analysing the results of the tests and model fitting:

1. River silt and cow dung powder were both incorporated into graphite tailings as organic matter amendments to improve their water-holding capacity. The Van Genuchten model fits the modified graphite tailings with an accuracy of no less than 97%, and the physical significance of the model fit parameters *α*, *m,* and *n* are also consistent with the experimental results. The SWCC results obtained from the graphite tailings before and after modification based on the combined tensiometer and filter paper method and Van Genuchten model fitting were accurate and appropriate.
2. After the matric suction reached the air entry value, the volumetric water content decreased rapidly with increasing matric suction, and the pore intake developed rapidly. With an increase in river silt doping, the air entry value increased and then decreased, whereas the number of internal macropores decreased and then increased. In the process of increasing the river silt doping to 30g, the modified graphite tailing specimens gradually condensed into agglomerate structure internally, the small agglomerate structure increased, the pore size and number became smaller, and the majority of the pore volume was occupied by gas. During this, the water film in the pores was difficult to discharge, and the water-holding capacity of the modified graphite tailings was enhanced.
3. With increasing river silt doping, the saturated water content gradually increased, the residual water content first increased and then decreased, and the water-holding capacity exhibits a trend of first increasing and then decreasing. Within the scope of the experimental study in this paper, the graphite tailings water holding capacity was optimal with 4g cow dung powder incorporation and 30g river silt incorporation.

## Data Availability

All data generated or analysed during this study are included in this published article.
